# Plant toxin β-ODAP activates integrin β1 and focal adhesion: A critical pathway to cause neurolathyrism

**DOI:** 10.1038/srep40677

**Published:** 2017-01-17

**Authors:** Rui-Yue Tan, Geng-Yan Xing, Guang-Ming Zhou, Feng-Min Li, Wen-Tao Hu, Fernand Lambein, Jun-Lan Xiong, Sheng-Xiang Zhang, Hai-Yan Kong, Hao Zhu, Zhi-Xiao Li, You-Cai Xiong

**Affiliations:** 1 State Key Laboratory of Grassland Agro-ecosystems, Institute of Arid Agroecology, School of Life Sciences, Lanzhou University, Lanzhou 730000, Gansu Province, China; 2 Department of Orthopaedics Surgery, General Hospital of Chinese People’s Armed Police Force, Beijing, 100039, China; 3 School of Radiation Medicine and Protection, Soochow University, Building 402 Room 2222, 199 Renai Road, Suzhou 215123, Jiangsu, China.; 4 Institute Plant Biotechnology for Developing Countries (IPBO), Department of Molecular Genetics, Faculty of Sciences, K.L. Ledeganckstraat 35, Ghent University, B-9000 Gent, Belgium

## Abstract

Neurolathyrism is a unique neurodegeneration disease caused by β-N-oxalyl-L-α, β- diaminopropionic (β-ODAP) present in grass pea seed (*Lathyrus stativus* L.) and its pathogenetic mechanism is unclear. This issue has become a critical restriction to take full advantage of drought-tolerant grass pea as an elite germplasm resource under climate change. We found that, in a human glioma cell line, β-ODAP treatment decreased mitochondrial membrane potential, leading to outside release and overfall of Ca^2+^ from mitochondria to cellular matrix. Increased Ca^2+^ in cellular matrix activated the pathway of ECM, and brought about the overexpression of β1 integrin on cytomembrane surface and the phosphorylation of focal adhesion kinase (FAK). The formation of high concentration of FA units on the cell microfilaments further induced overexpression of paxillin, and then inhibited cytoskeleton polymerization. This phenomenon turned to cause serious cell microfilaments distortion and ultimately cytoskeleton collapse. We also conducted qRT-PCR verification on RNA-sequence data using 8 randomly chosen genes of pathway enrichment, and confirmed that the data was statistically reliable. For the first time, we proposed a relatively complete signal pathway to neurolathyrism. This work would help open a new window to cure neurolathyrism, and fully utilize grass pea germplasm resource under climate change.

Neurolathyrism is a unique neurodegeneration disease caused by β-N-oxalyl-L-α, β- diaminopropionic (β-ODAP) in the seeds of grass pea (*Lathyrus stativus* L.) that has caused human and animal spastic paraparesis in China, India, Ethiopia and European countries over last one hundred years^1^ (see Supplementary Fig. S1). A recent large outbreak took place in Ethiopia in 1996, with over 2000 people affected in one village^2^,^3^. The symptoms are irreversible once the spasticity occurs. Unfortunately, none of the neurolathyrism cases is so far cured successfully or controlled pathologically^2^. While some efforts have been paid over the last decades, the critical signal pathway to cause this disease remained unclear. This restriction has become a major issue to take full advantage of grass pea as an elite germplasm resource under climate change and food security^4^,^5^,^6^. In-depth investigations on neurolathyrism pathogenesis have been largely lacked, while it should be a particularly important issue in multidisciplinary fields such as neurobiology, pathology, clinical medicine, biodiversity conservation, crop science and even global climate change biology.

Existing studies showed that neurolathyrism was caused by long-term overconsumption of grass pea seeds, containing up to 1% of β-ODAP in the seeds^7^. β-ODAP is actually a non-protein neuro-excitatory amino acid that can be considered glutamate analog. In comparison with the progresses achieved in general neurodegeneration biology over last decades, current understandings on the pathogenic mechanism of neurolathyrism are much limited. To date, very few studies were available to demonstrate the neuropathological changes following β-ODAP intake. Krogsgaard-Larsen *et al*. found that β-ODAP can act as an agonist of AMPA receptors, which resulted in accumulation of intracellular calcium ion up to a toxic level^8^. Saeed *et al*. reported that *in vitro* and *in vivo* treatments with β-ODAP can induce significant oxidative stress due to the interruption of electron transport chain in the mitochondria. Oxidative stress always excessively induced to produce reactive oxygen species (ROS) and mechanically mediated the neuron death^9^. Some evidences also showed that β-ODAP can inhibit the bioactivity of Xc^−^ as a competitive inhibitor of cysteine transporting system^10^, pass through the blood brain barrier, cause motor neuron injury and ultimately destroy central neuron system^11^. Jammulamadaka *et al*. postulated that β-ODAP might influence the MAPK pathway via down-regulation of phosphatidylethanolamine-binding protein 1(PEBP1)^12^. While there are currently a few experimental and clinical studies available, the relevant explorations are isolated and fragmented from each other. A relatively compete molecular pathogenesis of neurolathyrism is still not well understood.

As is well known, grass pea is an annual legume crop that has been domesticated during the Neolithicum era, and culturally used as a part of funeral offerings in the Egyptian pyramids. This long agronomic history may indicate the presence of traits that are beneficial to growers and consumers. Grass pea can withstand severe agro-climatic conditions such as drought, cold, soil infertility and water logging^13^,^14^,^15^. During drought-triggered famines, it becomes the cheapest and only food available, becoming survival food for the poor. Grass pea seeds are also a natural source for homoarginine, an alternative precursor for nitric oxide (NO) which is considered a stamina factor. In most regions with frequent drought and famine disasters, grass pea plays a critical role as the staple diet since it still has stable harvest and contains almost 30% protein in the seeds^16^,^17^. As mentioned above, β-ODAP is the primary neurotoxic component of *sativus* L., which acts as a critical role causing neurolathyrism. The symptom shows various degrees ranging from inability to walk without support to complete paralysis of lower limbs, and in extreme cases to death^3^. Theoretically, to take precautions against or treat this disease is much dependent on the progresses in neurolathyrism biology. Logically, neurolathyrism biology is relatively independent but closely related to neuroscience, particularly neurodegeneration biology. Previous studies were mainly aimed at clinical and immunohistochemical methods to reveal the pathogenesis of neurolathyrism such as the report by Singh *et al*.^18^. Singh *et al*. found the phenomenon regarding β-ODAP-mediated activation of calcium-dependent conventional protein kinase C (PKC) isozymes^18^. Yet, very few studies were aimed at gene expression pathways.

Currently, RNA-Seq is widely used to investigate pathological mechanism of neurodegeneration, such as Alzheimer’s Disease (AD)^19^ and Amyotrophic Lateral Sclerosis (ALS)^20^. It serves as an effective tool to analyze the sequencing variation in gene expression along with the progression of diseases. It is also used as a parallel sequencing method of transcriptome analysis to identify the transcribed portions of genome, and avoid the interference of non-coding and repetitive sequences that comprise much of the genome. On the basis of previous studies, we employed a few typical methods to make systematic investigations, including Ca^2+^ release, mitochondrial membrane potential, cell microfilament dynamics and gene sequencing and statistical analysis. The cell-line of human glioma cells M059K was used as test material to harvest the data and evidences. The results would provide direct evidences for main signal pathway of neurolathyrism pathogenesis, and new insight into intrinsic links between neurolathyrism and other neurodegeneration disorders.

## Material and Methods

### Cell Culture and β-ODAP Treatment

M059K cells were grown in DMEM/F12 with 5% fetal calf serum (FBS) at 37 °C in a 5% CO_2_ atmosphere. On the day before treatment, 1 × 10^5^ M059K cells in 5 mL cell culture medium without antibiotics were seeded onto a 60-mm dish to reach 80% confluence. β-ODAP and Glu were dissolved in double distilled H_2_O to the concentrations of 40 mM and 100 mM respectively. Particularly, β-ODAP and Glu were directly added to the media for a final concentration of 10 mM. Cells were collected after the treatment of 6 h, 12 h, 24 h, 48 h for further survey and observation.

### RNA Preparation and RNA-seq

Total RNA was isolated in 24 h after β-ODAP or Glu treatment using Trizol Reagent (MRC, Cincinnati, USA). In this study, total RNA from control cells was used as a reference. The samples of total RNA were first treated with DNase I to avoid any possible DNA contamination. Afterwards, the mRNA was enriched using the oligo (dT) magnetic beads (for eukaryotes). Following mixing with the fragmentation buffer, the mRNA was decomposed into short fragments (about 200 bp for each). Then the first strand of cDNA was synthesized by using random hexamer-primer. Buffer, dNTPs, RNase H and DNA polymerase I were added to synthesize the second strand. Subsequently, double strand cDNA can be purified with magnetic beads. End reparation and 3′-end single nucleotide A (adenine) addition was then performed. Finally, sequencing adaptors were ligated to the fragments. The fragments were further enriched by PCR amplification. During the QC process, Agilent 2100 Bioanaylzer and ABI Step One Plus Real-Time PCR System were employed to qualify and quantify the sample library. The library products were therefore sequenced via Illumina HiSeqTM 2000 (Illumina, Inc., San Diego, USA).

### Data Analysis

Millions of raw reads with a sequencing length of 35 bp were generated. The adaptors, empty tags (no tag sequence between the adaptors), low quality tags (tags containing one or more unknown nucleotides “N”) and tags with a copy number of 1 were removed from the raw data to obtain 21 bp clean tags. All clean tags were mapped to the transcriptome reference database generated by RNA-Seq. The number of unambiguous tags corresponding to each gene was calculated and normalized to the TPM (number of transcripts per million clean tags) to analyze the expression of different genes. The gene expression level is calculated by using RPKM^21^ method (Reads Per kb per Million reads), and the formula is shown as follows:


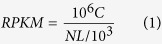


Here RPKM (A) is the expression level of gene A, C is the number of reads that uniquely aligned to gene A, N is total number of readings that uniquely aligned to all genes, and L is number of bases of gene A. The RPKM method is able to eliminate the influence of different gene length and sequencing discrepancy on the calculation of gene expression level. Therefore, the RPKM values can be directly used to compare the differences in gene expressions among samples. If there is more than one transcript for one gene, the longest one would be used to calculate its expression level and coverage.

Function annotation can provide Gene Ontology (GO) annotation information. GO covers three domains, including cellular component, molecular function and biological process. The basic unit of GO is GO-term. Every GO-term belongs to a type of ontology. GO enrichment analysis provides all GO terms that are significantly enriched in DEGs comparing with genome background, and can filter the DEGs that correspond to biological functions. This method firstly maps all DEGs to GO terms in the database (http://www.geneontology.org/), calculating gene numbers for every term and then using hypergeometric test, identifying significantly enriched GO terms in DEGs comparing with the genome background. The calculating formula is as follows:


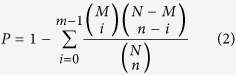


where N is the number of all genes with GO annotation, n is the number of DEGs in N, M is the number of all genes that are annotated to specific GO terms, m is the number of DEGs in M. The calculated p-value goes through Bonferroni Correction, taking corrected p-value ≤ 0.05 as a threshold. GO terms meeting this condition would be defined as significantly enriched GO terms in DEGs. This analysis is able to recognize the main biological functions that DEGs exercise. Pathway-based analysis helps further understand biological functions of all the genes. KEGG is the major public pathway-related database^22^. Pathway enrichment analysis identifies significantly enriched metabolic pathways or signal transduction pathways in DEGs comparing with the whole genome background. The calculating formula is the same as in GO analysis. Here N is the number of all genes with KEGG annotation, n is the number of DEGs in N, M is the number of all genes annotated to specific pathways, and m is the number of DEGs in M.

### qRT-PCR

Total RNA was isolated from the control cells and the β-ODAP or Glu treated cells after 6, 12, 24 and 48 hrs of treatment by the use of Trizol Reagent (MRC, Cincinnati, USA). The cDNA was synthesized from 1 μg DNase I-treated RNA using the synthesis kit (Promega, Madison, WI, USA). The cDNA solution was diluted to 1:5 with nuclease-free water. Real-time PCR was performed using supermix (Promega, Madison, WI, USA) and specific primer pairs for the selected genes (Primers seq isshown in Table 1), the primers for GAPDH as reported previously^23^. For each sample, the following mix was prepared: 6 μL cDNA, 30 μL Supermix, 100 nM of each primer in nuclease-free water to a final volume of 60 μL. Aliquots of 20 μL were distributed in three wells on the PCR plate. Real-time PCR reactions were run on a CFX96 (Bio-Rad) following amplification protocol: 10 min initial denaturation at 95 °C, 44 cycles of 10 s denaturation at 95 °C, 15 s annealing at 58 °C and 15 s extension at 72 °C. A final melt curve analysis was included to verify that a single specific product was obtained in each reaction. Expression quantification and data analysis were performed in accordance with Bustin *et al*.^24^. Significant differences in expression levels among different samples and time points were determined by a student’s t test using SPSS software (version 13.0; SPSS, Inc., Chicago, IL, USA). A probability (*p*) value of < 0.05 was considered statistically significant.

### Flow Cytometry

The fluorescent probe Rho123 was used to measure the membrane potential of mitochondria. Untreated cells and cells treated with β-ODAP or Glu for 6, 12, 24 and 48 hrs were collected. Cell monolayers were washed with experimental medium (DMEM containing 20 mM HEPES, pH 7.4 and 0.1% BSA) and then incubated in this medium with the fluorescent probes at 37 °C under 5% CO_2_ for the indicated lengths of time. Cells were added to the monolayers either before or after loading the probes, as indicated. At the end of the incubations, cells were washed twice with cold PBS, and resuspended by trypsinization at room temperature with 0.5 mL trypsin/EDTA per well. After adding 0.5 mL trypsin neutralizing solution, the cell suspension was stored in the dark at 4 °C until the time of analysis (usually within 30 min). PI (2.5 mg/mL) was added to cell suspensions stained with Rho123 to identify dead cells. Flow cytometry was performed on a FACSs instrument (Becton Dickinson, Oxford, UK) which was connected to an Apple G3 Computer. Data were harvested and analyzed using Cell Quest software (Becton Dickinson). The analyzer threshold was adjusted on the FSC channel to exclude noise disturbance and most of subcellular debris. Photomultiplier settings were adjusted to detect Rho123 fluorescence on the FL1 detector, and PI fluorescence on the FL3 detector. In each case, the photomultiplier voltage was set so that the signal peak from non-stained cells (mostly due to autofluorescence) can fall within the first decade of the logarithmic amplifier. The linearity of logarithmic amplifiers was checked routinely using standardized fluorescent beads (Immunobrite, Coulter). Light scatter parameters were used to establish size gates and detect apoptotic cells^25^.

### Immunofluorescence and Confocal Microscopy

Cells were cultured at low density on coverslips to ensure log phase growth and be treated with β-ODAP or Glu for 6 hrs and 24 hrs. For co-loading with fluo-3 and Mitotracker^®^ Red M7512, the cells were incubated for 30 min at 37 °C with 0.1 μM- Mitotracker M7512, 1 μM fluo-3 AM and 3 μM Hoechst 33342 in the dark. Hoechst 33342 was used for locating the nucleus. In all experiments, a control sample of cell suspension incubated in the absence of dye was used. For fluorescence measurements, fluo-3 AM (505 nm excitation), M7512 (579 nm excitation) and Hoechst 33342 (346 nm excitation) were measured with Zeiss LSM510 laser scanning confocal microscope (Carl Zeiss Ltd.).

### F-actin Assay

Cells were grown on coverslips and treated with β-ODAP or Glu for 6, 12, 24 and 48 hrs. The medium was removed and 1 ml of 3.7% (v/v) paraformaldehyde in PBS was added for 5 minutes, then permeabilized with 1 ml of 0.1% (v/v) Triton X-100 in PBS for 10 minutes. Staining with 50 μg/mL fluorescent, phalloidin conjugate solution in PBS (containing 1% DMSO from the original stock solution) was added and incubated for 40 minutes at room temperature. The cells were then rinsed for 3 times with PBS as mentioned above. About 5 μL of 10 mg/mL 4′-6-diamidino-2-phenylindole (DAPI; Sigma) in PBS was added to each glass slide, and the slides were imaged using a Zeiss LSM510 laser scanning confocal microscope (Carl Zeiss Ltd.).

## Results

### Digital Gene Expression (DGE) Profiles

To investigate the changes in whole gene expression following β-ODAP/Glu treatments, individual DGE tag libraries were established from 3 total RNA samples isolated from the β-ODAP/Glu and control groups, and then sequenced by Illumina HiSeq 2000. Approximately 25 million raw tags were generated for each DGE library, and more than 99% of raw tags were clean in each library.

Samples were treated for 24 hrs using Glu and compared with those of control group. The pairs of DGE profiles were analyzed to identify differentially-expressed genes (DEGs). It showed no significant differences in DEGs between two treatments. We further compared the differences in DGE between β-ODAP-treated and control groups. Significant differences in DGE ratios were observed, ranging from −2.23 to 4.72 in β-ODAP treatment group. Specifically, 72 unigenes were identified to be in up-regulation while 63 unigenes in down-regulation. Top 10 up- and down-regulated genes are listed in the supplemental documents.

### Gene Ontology (GO) Term Enrichment Analysis on differentially Expressed Genes

**Figure 1:**
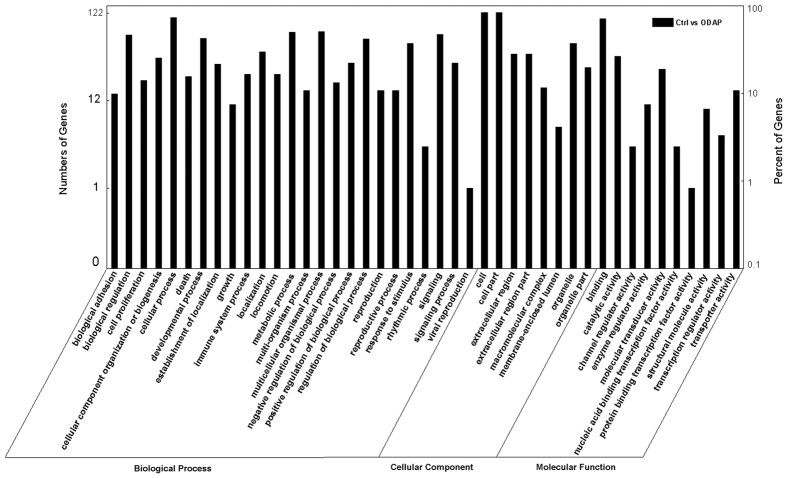
Gene Ontology analysis of DEGs in group under β-ODAP treatment.

GO analysis indicated that most of DEGs could be grouped into 3 categories including *Biological Process, Cellular Component* and *Molecular Function* with total 43 clusters inside (Fig. 1). There were 25, 8 and 10 clusters in *Biological Process, Cellular Component* and *Molecular Function* respectively. Among 25 clusters in *Cellular Process*, 5 clusters including biological regulation, developmental process, metabolic process, multi cellular organismal process and signaling were found to have higher expression proportion of DEGs. Among 8 clusters in *Cellular Component* category, 3 clusters including cell, cell part, and organelle accounted for a higher proportion. As for *Molecular Function*, 3 clusters including binding, catalytic activity, molecular transducer activity had a higher proportion of expression than other 7 ones. Further component ontological analyses indicated that extracellular region was observed to have the most enriched clusters after β-ODAP treatment (Fig. 1). The genes including *fibronectin 1, MSTN* and*CRLF1* were up-regulated at least two-fold expression level while the genes including *SLPI, complementcomponent3* and *amphiregulin* were identified in down-regulation. For other clusters with relatively low proportion of DEGs expression, there were detectable changes from intracellular to cell membrane, most of which were associated with cell signal transduction (see Supplementary Table S2). The changes mainly took place on receptor binding and ion channels. For example, the genes like *COMP, LAMC2, MSTN, CACNA2D3* were up-regulated while *CD74, TGFA, KCNK5* were down-regulated (see Supplementary Table S2). Receptors and ion channels played critical roles in cross-cell signal transduction. The results of GO analyses indicated that β-ODAP could modify the signal transduction process through changing the expression level of key genes in 3 categories of DEGs (Fig. 1).

### Highlighted KEGG Pathways of β-ODAP Treatment

**Figure 2:**
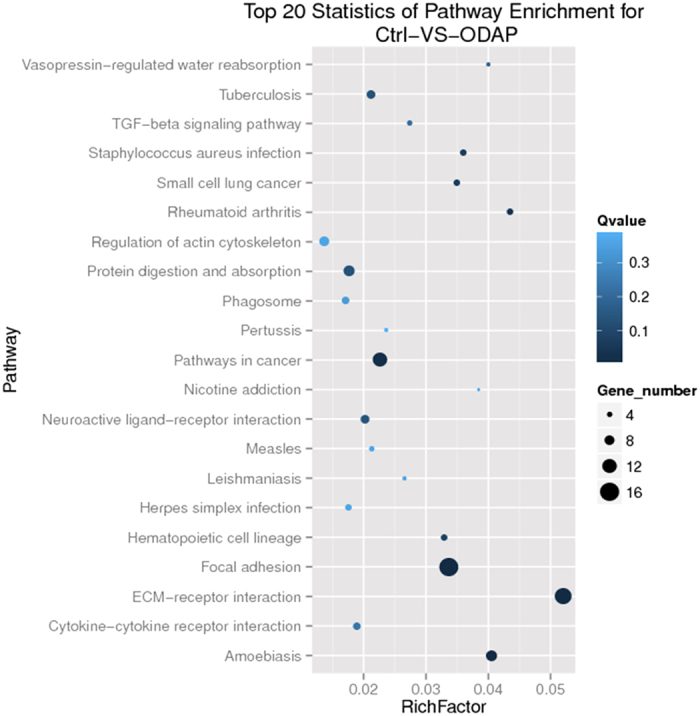
The top 20 statistics of pathway enrichment for group under β-ODAP treatment.

Kyoto Encyclopedia of Genes and Genomes (KEGG) is the major public pathway-related database^21^. Pathway enrichment analysis was made to identify significantly enriched metabolic pathways or signal transduction pathways in DEGs under whole genomic background. DEGs with KEGG annotations were allocated in 140 KEGG pathways, most of which were linked with signal transduction or cell junction. The top 20 statistics of pathway enrichment for β-ODAP treated samples is shown in Fig. 2.

There existed 14 DEGs in extracellular matrix (ECM) interaction pathway, 12 of which were up-regulated while 2 were down-regulated. The expressions of genes including *Laminin, THBS, Fibronectin, Tenascin, integrin* were wholly enhanced as a product of β-ODAP treatment. However, some collagen genes were suppressed by β-ODAP. There were 16 DEGs in focal adhesion pathway, which were allocated at the downstream of ECM receptor interaction pathway. *Integrinβ1* gene was significantly upregulated following β-ODAP treatment, and in the meantime *paxillin* gene was downstream overexpressed. The expression levels of other genes such as *MLC, Vav, MLCP* were changed at a certain degree. Therefore, the genes as presented in above pathways were assiociated with actin polymeration. It can be argued that β-ODAP treatment could induce the differentiate expressions of the genes involved in cytoskeleton dynamics (Fig. 2).

### Validation on DGE Tag Data using qRT-PCR Expression Pattern

**Figure 3:**
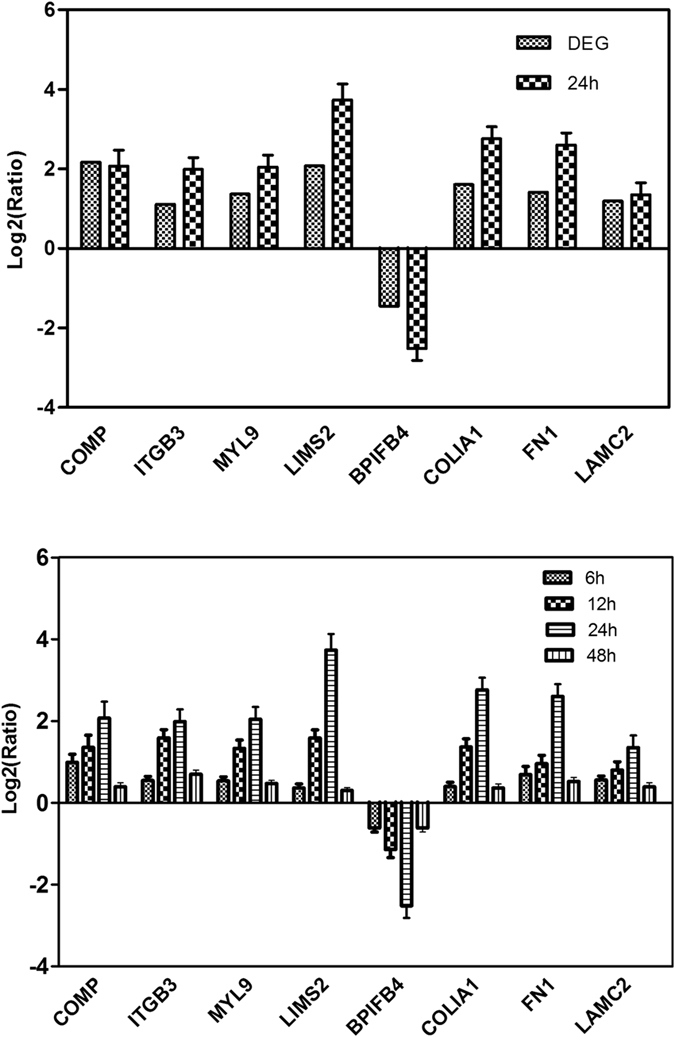
Verification of RNA-seq results using the RT-qPCR analysis, qRT-PCR to analysis of DEGs in different time points post β-ODAP treatment.

In order to validate our DGE data, eight unigenes with annotations were randomly chosen to receive the test of qRT-PCR Expression Pattern. The results showed that the qRT-PCR data regarding these genes were consistent with those of DGE results (Fig. 3). For example, both qRT-PCR and DGE analyses showed that the encoding *FN1* and *COMP* genes were expressed at significantly higher level in the β-ODAP-treated group than those of control group. Likewise, the expression of *BIBPF4* gene was suppressed by β-ODAP treatment, which confirmed the results of DGE analysis verified with qRT-PCR analysis (Fig. 3).

In order to confirm if treatment duration would influence the expression levels of genes, we further analyzed the samples after 6 h, 12 h and 48 h of β-ODAP treatment. The expression of eight genes displayed the same trends at four time points (Fig. 3). The results further confirmed that β-ODAP had an increasing up- or down-regulation influence on DGE with the extension of treatment time.

### Dynamics of Ca^2+^ in Mitochondria and Cell Matrix

**Figure 4:**
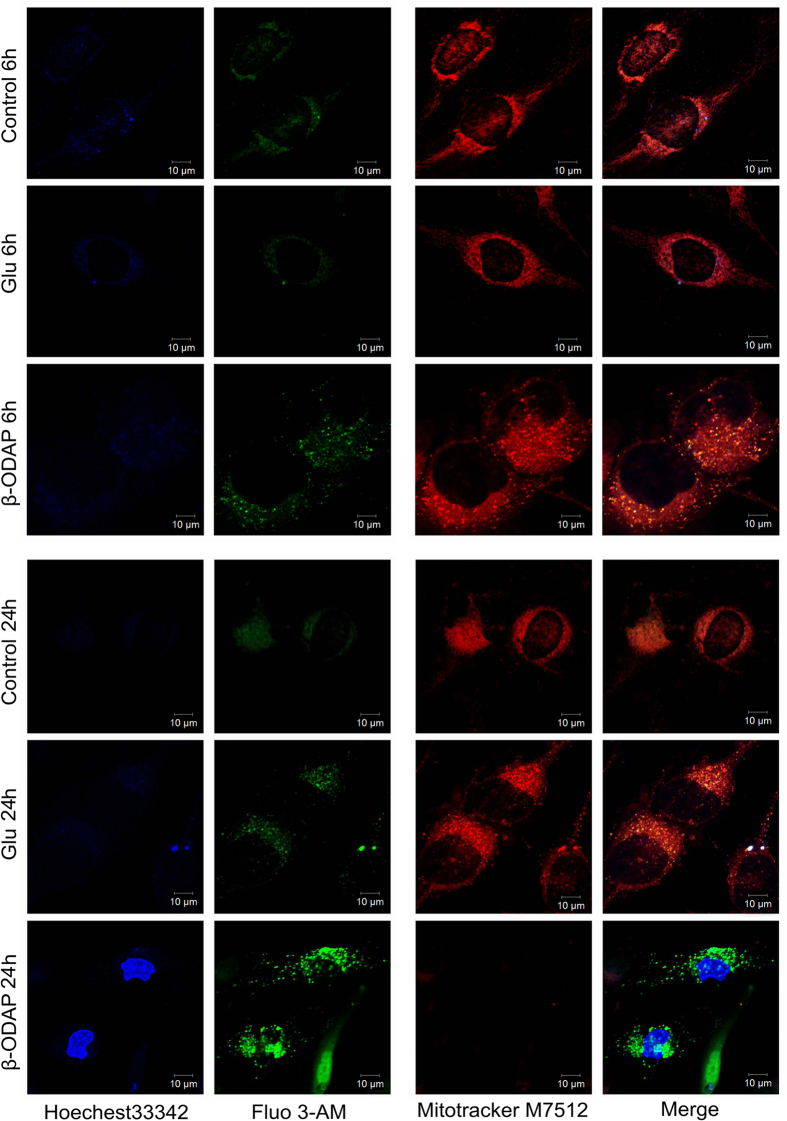
Dynamic of Ca^2+^ and mitochondria activity for cells response to β-ODAP or Glu treatment in 6 h and 24 h.

We used fluorescence imaging with fluo-3, a (Ca^2+^)i indicator dye, to measure (Ca^2+^)i in M059K cell lines. The (Ca^2+^)i started to increase at 6^th^ hour after β-ODAP treatment, whereas there was not significant increase in control cells and the Glu-treated ones (Fig. 4). After 24 hrs of β-ODAP treatment, the intensity of Fluo-3 AM fluorescence was enhanced significantly as compared to 6 h treatment. What’s more, the (Ca^2+^)i was increased as well after 24 hrs of Glu treatment (Fig. 4).

In order to find out if the Ca^2+^ in cytoplasm was localized at mitochondria, we used the Mito Tracker M7512 for co-localization. After 6 hrs of β-ODAP treatment, most of arising Ca^2+^ was co-localized at mitochondria (Fig. 4). Interestingly, the fluorescence of Mito Tracker M7512 could not be observed after 24 hrs of β-ODAP treatment. It suggested that the loss of mitochondrial integrity be induced by over influx of Ca^2+^ (Fig. 4).

### Evaluation of Mitochondrial Membrane Potential

Traditional mitochondrial probe rhodamine-123 has been widely used to evaluate mitochondrial membrane potential (Δ*ψ*_m_) in a variety of cell types. We explored the effect of β-ODAP treatment on Δ*ψ*_m_ as measured in M059K cells. The cells treated with both β-ODAP or Glu (10 mM, 12 h) showed no significant changes in mitochondrial potential. However, after 24 hrs of treatment, β-ODAP group showed an obvious fluorescence enhancement while the Glu group did not have detectable changes. Furthermore, after 48 hrs of treatment, the same results were observed (Fig. 4). Thus, the observations demonstrated that β-ODAP played a critical role in decreasing the membrane potential Δ*ψ*_m_ of mitochondria.

### Dynamics of Cytoskeleton Polymeration

**Figure 5:**
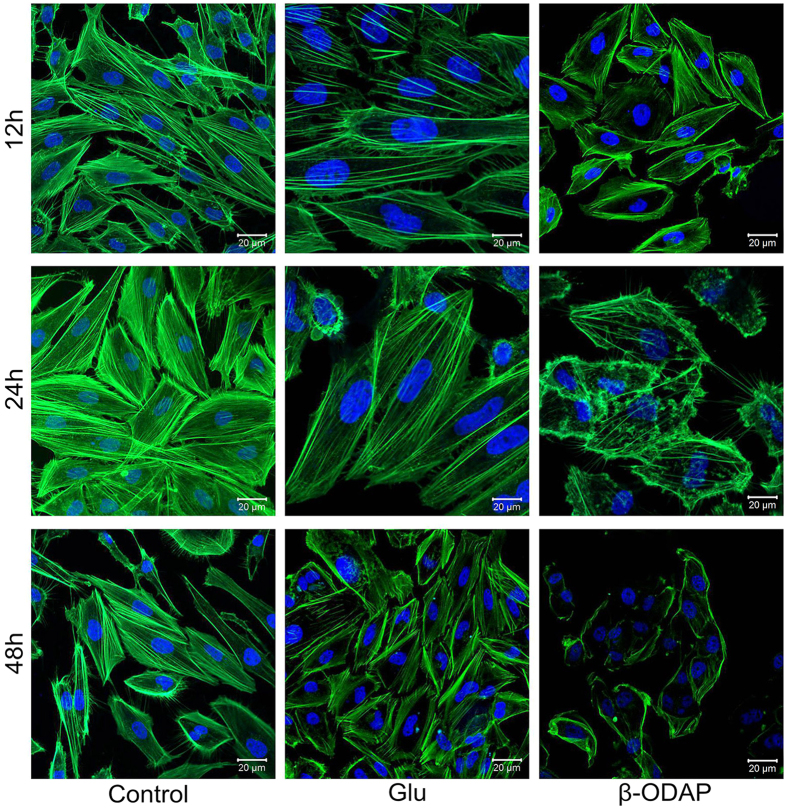
Cytoskeleton dynamic of cells respons to β-ODAP or Glu treatment in different time points.

The KEGG analysis indicated that most of DEGs in FA pathway were much associated with the activities of cytoskeleton (Fig. 2). In order to verify the effects of β-ODAP treatment in this aspect, we employed the method of paraformaldehyde to further track the changes in microtubule and actin, as they were major proteins of cytoskeleton to adjust cell configuration. The M059K cells were treated with 10 mM β-ODAP and Glu respectively for 12–48 hrs. The result showed that β-ODAP treatment reduced the fluorescence intensity of F-actin after 12, 24 and 48 hrs, while Glu treatment did not indicate a similar trend until the 48^th^ hour. Importantly, the disorder of microtubule was clearly observed after 24 and 48 hrs of β-ODAP treatment, whereas there was not similar phenomenon at three time points of Glu treatment (Fig. 5). These results suggested that β-ODAP treatment had a negative regulatory effect on actin polymeration.

## Discussion

Grass pea as a drought-tolerant legume crop is extensively planted in many drought-prone areas of world, preferentially semiarid areas with unreliable rainfall^1^. Domesticated in the Neolithic era, it has retained its popularity till present, apparently because of agronomic and sensory qualities. Grass pea is used for food, feed and forage, or for improving soil fertility. It is considered as a famine-relieving and life-saving food source that has saved thousands of human lives during droughts in Ethiopia, India, Bangladesh, China and other developing countries. However, neurolathyrism epidemics have given the plant a toxic reputation, making it a cheap food mostly for the poor^26^. In this case, better understanding on pathological mechanism of neurolathyrism would lead to a very broad perspective to cure the disease and to fully utilize the germplasm resources under the increasing shrink of crop genetic resources.

Since 1960 s, a large number of efforts have been paid on investigating the potential role of β-ODAP as a glutamate analog and its interaction with glutamate receptors and neuron signaling. Unfortunately, little progress has been so far achieved at cellular level in term of molecular mechanism of neurolathyrism pathology. Particularly, there has been still very limited information available regarding its molecular mechanism. In this study, we incorporated our experimental evidences into previous studies in order to sketch the general pathway of molecular mechanism. For the first time, our study provided a relatively comprehensive insight into the transcriptome of neurodegeneration induced by β-ODAP. Using a whole transcriptome sequencing technique (RNA-Seq), we were therefore able to identify the genetic variation at the levels of differentially expressed genes (DEGs) when exposed to β-ODAP treatment. The results might help find an effective pathway to control human neurolathyrism efficiently.

In virtue of the unigene reference dataset, our DGE analysis provided clear observational evidences for understanding on molecular mechanism at the cellular level (Table S1). Systematic analysis on DEGs and Gene Ontology term enrichment showed that β-ODAP played a critical role in the regulation of cellular signal transduction and physiological development. As shown in Fig. 2, enriched KEGG pathways indicated that several typical biological processes were highlighted in molecular expression in the β-ODAP treatment group. The most highlighted pathways were extracellular matrix (ECM)-receptor interaction pathway as well as focal adhesion pathway. Existing studies suggested that ECM can act as an important regulator influencing many aspects of cellular function. Especially, specific ECM molecules tended to bind with integrin cell surface receptors and then activate downstream focal adhesion, which contributed much to cell junction and signal transduction^27^. As previously studied in brain neuron, integrins and numerous FA CAMs can be expressed and activated throughout central nervous system.

**Figure 6:**
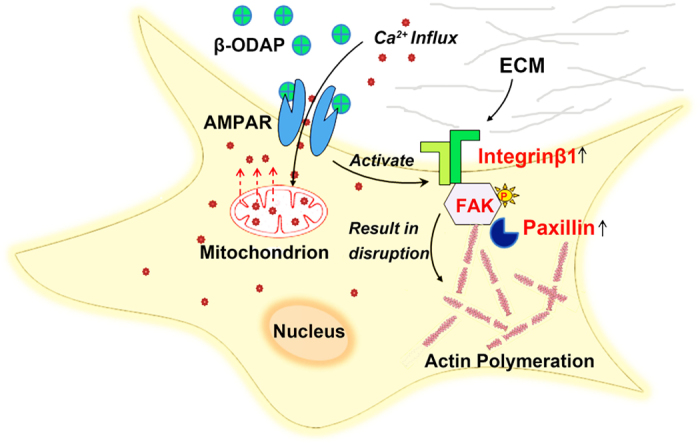
The mechanism of decay induced by β-ODAP. The combination of β-ODAP and over-activating AMPA receptors triggers the signaling cascade of reactions with intracellular Ca^2+^ flow. Meanwhile, increased expression level of β1 integrin on the cell surface will substantially induce the phosphorylation level of FAK and overexpression of paxillin. This will cause massive aggregation of FA units on cell actin filaments, seriously interfere the assembly of cell microfilaments, and ultimately result in structural damage of the cells.

In the realm of neurodegeneration research, numerous studies indicated that integrin signaling played a key role in neurite outgrowth in response to toxic effects associated with neurodegeneration^28^,^29^. Recent reports showed that if fibrillar Aβ was added to cells in culture, both FAK and paxillin would be rapidly over phosphorylated, leading to downstream signaling events and neuronal dystrophy^30^. It can be concluded that the series of reactions should be initiated following the activation of β1 integrin. In neuron system, the activation of AMPA receptors can increase surface expression of β1 integrin, subsequently activate the FA pathway, and ultimately adjust cell migration and interaction^31^. Since β-ODAP is a strong agonist of AMPA receptors, it is very possible to produce neurodegenerative phenomenon through the excitotoxic interaction with AMPA receptors^32^. In our study, the significant up-regulation of β1 integrin was observed (Fig. 6). This process was likely mediated via abnormal activation of AMPA receptors as a result of β-ODAP induction (Figs 2 and 6). Piao *et al*. found that AMPA receptors acted as membrane-associated cytoskeleton anchors to localize signaling complexes at FAs^33^,^34^.

More evidences showed that excessive activation of AMPA receptors would cause intracellular Ca^2+^ flow, enhance the expression level of β1 integrin on the cell surface, and accordingly raise the phosphorylation level of focal adhesion kinase (FAK)^33^. Other studies on the mechanism of neural injury towards Alzheimer disease (AD) showed that abnormal activation of FA protein units would result in massive accumulation of paxillin (widely recognized as an important component of FA) on cytoskeleton, which further caused the hyperphosphorylation of Tau and the injury of cytoskeleton, and ultimately induced nutrition disorder of neuron. As identified in previous study, Tau hyperphosphorylation was one of the central events in the pathogenesis of AD^28^.

In present study, there was a significant increase in expression level of β1 integrin in FA pathway following β-ODAP treatment, and the overexpression of paxillin in the downstream of this pathway subsequently occurred. In earlier studies, it has been reported that β-ODAP was able to combine with and activate AMPA receptors^32^. Thus, we postulate that β-ODAP combining with and over-activating AMPA receptors can trigger the signaling cascade of reactions with intracellular Ca^2+^ flow and ultimately result in cellular dysfunction. In this case, expression level of β1 integrin on the cell surface would be largely increased, and accordingly phosphorylation level of FAK would be further raised as well. Thus, FA protein units were massively aggregated on cell actin filaments, leading to overexpression of paxillin. Furthermore, excessive accumulation of paxillins turned to seriously interfere the assembly of cell microfilaments, resulting in structural damage of the cells, and ultimately causing neuron nutrition disorder. Our study demonstrated a potentially novel mechanism to account for nerve cell damage induced by β-ODAP (Fig. 6).

On the basis of our experimental observations and other colleagues’ previous studies, we attempted making a relatively in-depth discussion on the pathogenesis of β-ODAP-induced neurolathyrism. As is well known, the formation of FAs is positively correlated with the quantity of paxillin. We found that increased expression of paxillin after β-ODAP treatment brought about the increases in FAs, suggesting that β-ODAP treatment had a strong activating effect on FA signaling cascades. At tissue level, FAs including FAK and paxillin played a critical functional role in regulating cell adhesion and survival during the process of neuronal differentiation^28^. It is noted that aberrant activation of FA proteins is a main driving force to cause the dystrophy of neuronal cells, which mainly results from inefficient actin polymeration^30^. Our study provided the observational evidences about microtubule disorder after β-ODAP treatment for 12 hrs, confirming the phenomenon of aberrant activation of FA pathway. Therefore, the expression of β1 integrin should be up-regulated after β-ODAP was combined with AMPA receptors. During this process, the related FA units were excessively formed. Hence, aberrant activation of FAs might be a critical step to cause the disintegration of microfilament and microtubule, and ultimately impel the dystrophy of motorneurons (Fig. 6).

Other evidence suggested that (Ca^2+^)i accumulation played a critical role in the process of β-ODAP induced neurolathyrism^35^. The toxin was a potent agonist of AMPA-type glutamatergic receptor towards Ca^2+^-impermeable and Ca^2+^-permeable subtypes^36^. The activation of AMPA receptors was considered as a major step to induce motor neuronal death mediated by the Ca^2+^-permeable-AMPA receptor^37^. In present study, the concentration of Ca^2+^ in cellular matrix was measured using Fluo-3 AM. The fluorescence intensity was increased at 6^th^ hour and further boosted at 12^th^ hour following β-ODAP treatment. In the meantime, there were only slight changes in fluorescence intensity after Glu treatment. At 6^th^ hour of β-ODAP treatment, most of Ca^2+^ were co-localized on the mitochondria. It can be argued that Ca^2+^ would influx into cytoplasmic matrix and be gradually absorbed by mitochondria. This idea was supported by the observational evidence that mitochondrial membrane potential tended to decrease after β-ODAP treatment. It was noted that at 24^th^ hour of β-ODAP treatment, the fluorescence of Mito-Tracker M751 cannot be detected. It was likely that Ca^2+^ over-absorption by mitochondria led to the loss of function of mitochondria (Fig. 4). Existing knowledge agrees that abnormal increase in Ca^2+^ can suppress the actin polymeration via the activation of calmodulin (CaM).

It can be concluded that β-ODAP can combine with AMPA receptors on cell surface and then abnormally activate the receptors, thereafter influencing intracellular Ca^2+^ flow and destroying intracellular homeostasis. Under such circumstance, cell viability rate tended to decline rapidly and excessive intake of Ca^2+^ into mitochondria altered mitochondrial membrane potential. This in turn blocked ATP synthesis, increased ROS production, damaged biological membrane, and ultimately resulted in cell dysfunction and death. On the other hand, the activated AMPA receptors would enhance β1integrin expression, and further raise the FAK phosphorylation level. FA protein units would massively aggregate on cell actin filaments, induce the increase in paxillin expression, suppress the polymerization of cytoskeleton, and finally cause cellular nutrition disorder and injury to nervous system (Fig. 6).

In summary, we attempted constructing a genome-wide transcript profile regarding β-ODAP-induced neurolathyrism by the methods of incorporating our experimental observations into other researchers’ previous work. Cell decay was a result of aberrant activation of FA pathway, which was mechanically meditated by β-ODAP-induced abnormal activation towards AMPA receptors. This insight help enhance the understanding on pathological mechanisms of neurolathyrism. Particularly, over influx of calcium led to disequilibrium of intracellular calcium homeostasis, gave rise to oxidative stress, accordingly caused mitochondria injury and ultimately induced the damage or death of cells. Taken together, it was a critical pathway for plant toxin β-ODAP to cause neurolathyrism by activating integrin β1 and focal adhesion. This study provided a relatively complete insight into molecular mechanisms of neurolathyrism. This work would help open a new window to cure or control the neurolathyrism, and also find a solution to take full advantage of grass pea as elite germplasm resource under climate change. In addition, β-ODAP treatment could serve as a potential effective manner or tool for the similar studies on neurodegeneration. This would help provide a novel approach for future investigations on pathological mechanisms of other neurodegenerative diseases.

## Additional Information

**How to cite this article**: Tan, R.-Y. *et al*. Plant toxin β-ODAP activates integrin β1 and focal adhesion: A critical pathway to cause neurolathyrism. *Sci. Rep.* **7**, 40677; doi: 10.1038/srep40677 (2017).

**Publisher's note:** Springer Nature remains neutral with regard to jurisdictional claims in published maps and institutional affiliations.

## Supplementary Material

Supplementary Information

## Figures and Tables

**Table 1:** Primers using for realtime PCR.

Name	Gene	Polarity	Sequence (5′ to 3′)
Y101	COMP	Sense	CTTCAGGGCCTTCCAGACAG
		Antisense	TCGAAGTCCACGCCATTGAA
Y102	ITGB3	Sense	CAGCAATGTCCTCCAGCTCAT
		Antisense	GAAGCTCACCGTGTCTCCAATC
Y103	MYL9	Sense	GCCCATCAACTTCACCATGTTC
		Antisense	TCCTCATCTGTGAAGCGGTCA
Y104	LIMS2	Sense	AGCCATGTGATTGAAGGCGA
		Antisense	CACCTCTTACACACGGGCTT
Y105	BPIFB4	Sense	CCTCAGGGTGACGAAAGATGTGT
		Antisense	GTAGGGAATATCACCAACACCCAA
Y106	FN1	Sense	TGCCAAAGCTTTACTACTGTGGA
		Antisense	ATTTCCCCCGAAGGTGTCTTATAA
Y107	COL1A1	Sense	GCGGGAGAGACTGTTCTGTTC
		Antisense	CCACCCCACCCATCACATAGAT
Y108	LAMC2	Sense	CGCAGAGTGAGAACCACCAA
		Antisense	ACTGCCTGGACTTCCCATTG
Y109	GAPDH	Sense	ACCCAGAAGACTGTGGATGG
		Antisense	TCTAGACGGVAGGTCAGGTC
