# Supplementary material for: Plant toxin β-ODAP activates integrin β1 and focal adhesion: A critical pathway to cause neurolathyrism
Source: Sci Rep. 2017 Jan 17;7:40677. doi: 10.1038/srep40677 (PMC5240565; doi:10.1038/srep40677)
Supplement: Supplementary Information [file srep40677-s1.doc]

**Plant toxin β-ODAP activates integrin β1 and focal adhesion: A critical pathway to cause neurolathyrism**

**Rui-Yue Tan^a^, Geng-Yan Xing^a,b^, Guang-Ming Zhou^c^, Feng-Min Li^a^, Wen-Tao Hu^e^, Fernand Lambein^d^, Jun-Lan Xiong^a^, Sheng-Xiang Zhang^a^, Hai-Yan Kong^a^, Hao Zhu^a^, Zhi-Xiao Li^a^, You-Cai Xiong^a,1^**

^a^ State Key Laboratory of Grassland Agro-ecosystems, Institute of Arid Agroecology, School of Life Sciences, Lanzhou University, Lanzhou 730000, Gansu Province, China.

^b^ Department of Orthopaedics, General Hospital of Armed Police Force, Beijing, 100039, China

^c^ School of Radiation Medicine and Protection, Soochow University, Building 402 Room 2222, 199 Renai Road, Suzhou 215123, Jiangsu, China.

^d^ Institute Plant Biotechnology for Developing Countries (IPBO), Department of Molecular Genetics, Faculty of Sciences, K.L. Ledeganckstraat 35,Ghent University, B-9000 Gent, Belgium

^e^ Gansu Key laboratory of Space Radiobiology / Key Laboratory of Heavy Ion Radiation Biology and Medicine, Institute of Modern Physics, Chinese Academy of Sciences, 509 Nanchang Road, Lanzhou 730000, China

Table S1. The top 10 up- and down-regulated genes

| GeneID | Symbol | Up-Down-Regulation | P-value |
| --- | --- | --- | --- |
| 128414 | NKAIN4 | Up | 7.40E-14 |
| 93659 | CGB5 | Up | 1.15E-40 |
| 1E+08 | FLJ16779 | Up | 7.48E-08 |
| 3861 | KRT14 | Up | 8.46E-06 |
| 4648 | MYO7B | Up | 1.34E-14 |
| 11174 | ADAMTS6 | Up | 3.52E-133 |
| 1311 | COMP | Up | 4.07E-07 |
| 3779 | KCNMB1 | Up | 1.52E-16 |
| 55679 | LIMS2 | Up | 2.29E-07 |
| 9244 | CRLF1 | Up | 7.06E-12 |
| 11001 | SLC27A2 | Down | 0.000102 |
| 150372 | NFAM1 | Down | 0.000102 |
| 5999 | RGS4 | Down | 6.80E-11 |
| 55273 | TMEM100 | Down | 2.37E-15 |
| 130576 | LYPD6B | Down | 9.62E-11 |
| 26074 | C20orf26 | Down | 2.78E-08 |
| 51129 | ANGPTL4 | Down | 5.17E-06 |
| 51393 | TRPV2 | Down | 2.09E-05 |
| 1356 | CP | Down | 7.05E-13 |
| 441478 | NRARP | Down | 1.07E-05 |
| 259232 | NALCN | Down | 2.80E-13 |

Table S2. The representative up- and down-regulated genes in Gene Ontology (GO) Term Enrichment Analysis

| GeneID | Symbol | Up-Down-Regulation | P-value |
| --- | --- | --- | --- |
| 1311 | COMP | Up | 4.07E-07 |
| 9244 | CRLF1 | Up | 7.06E-12 |
| 2660 | MSTN | Up | 1.36E-07 |
| 2335 | FN1 | Up | 0 |
| 3918 | LAMC2 | Up | 5.86E-10 |
| 55799 | CACNA2D3 | Up | 6.47E-07 |
| 7039 | TGFA | Down | 2.78E-22 |
| 8645 | KCNK5 | Down | 4.22E-06 |
| 6590 | SLPI | Down | 4.02E-36 |
| 718 | C3 | Down | 3.79E-149 |
| 374 | AREG | Down | 2.39E-10 |
| 972 | CD74 | Down | 8.72E-07 |


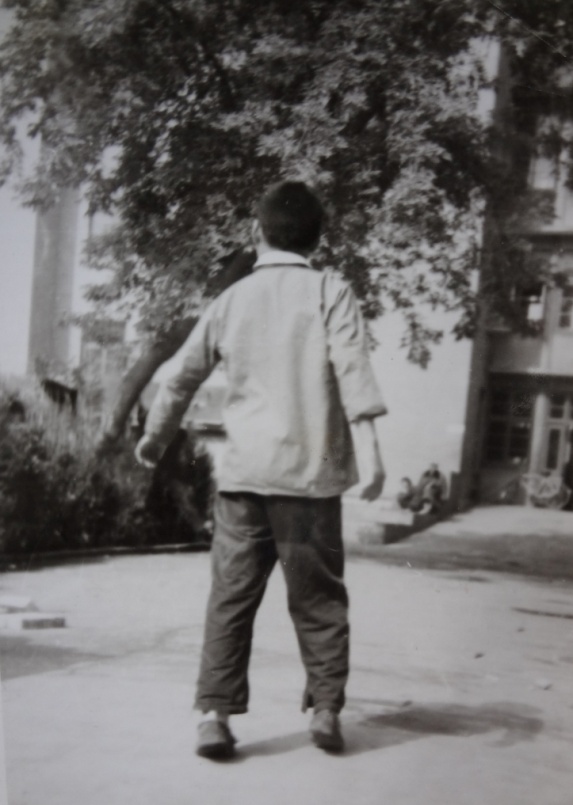


**Fig. S1. The neurolathyrism caused by over consumption of *Lathyrus Sativus* L. seeds**


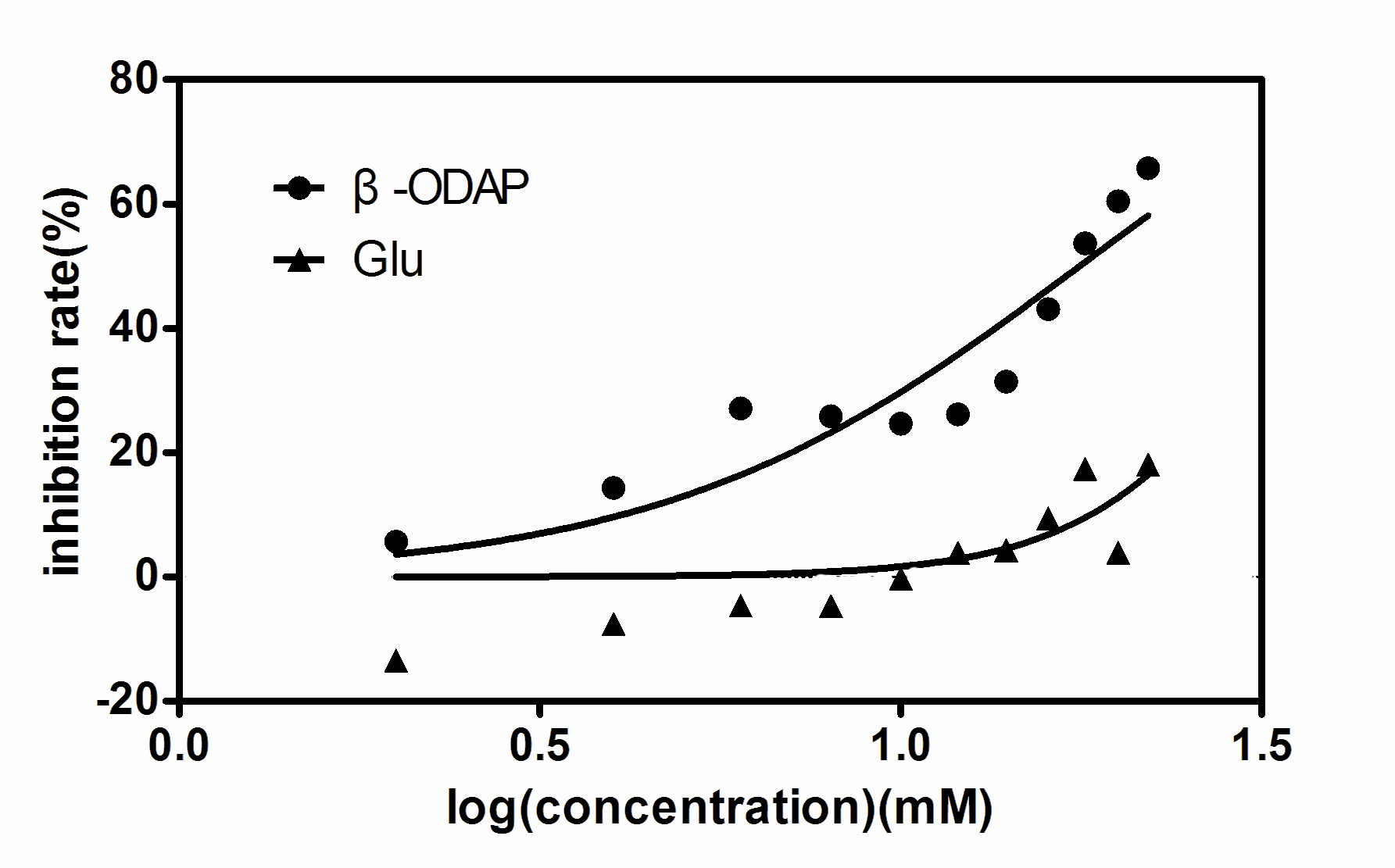


**Fig. S2. Dynamics of cell inhibition rates in response to different concentrations of Glu and β-ODAP**


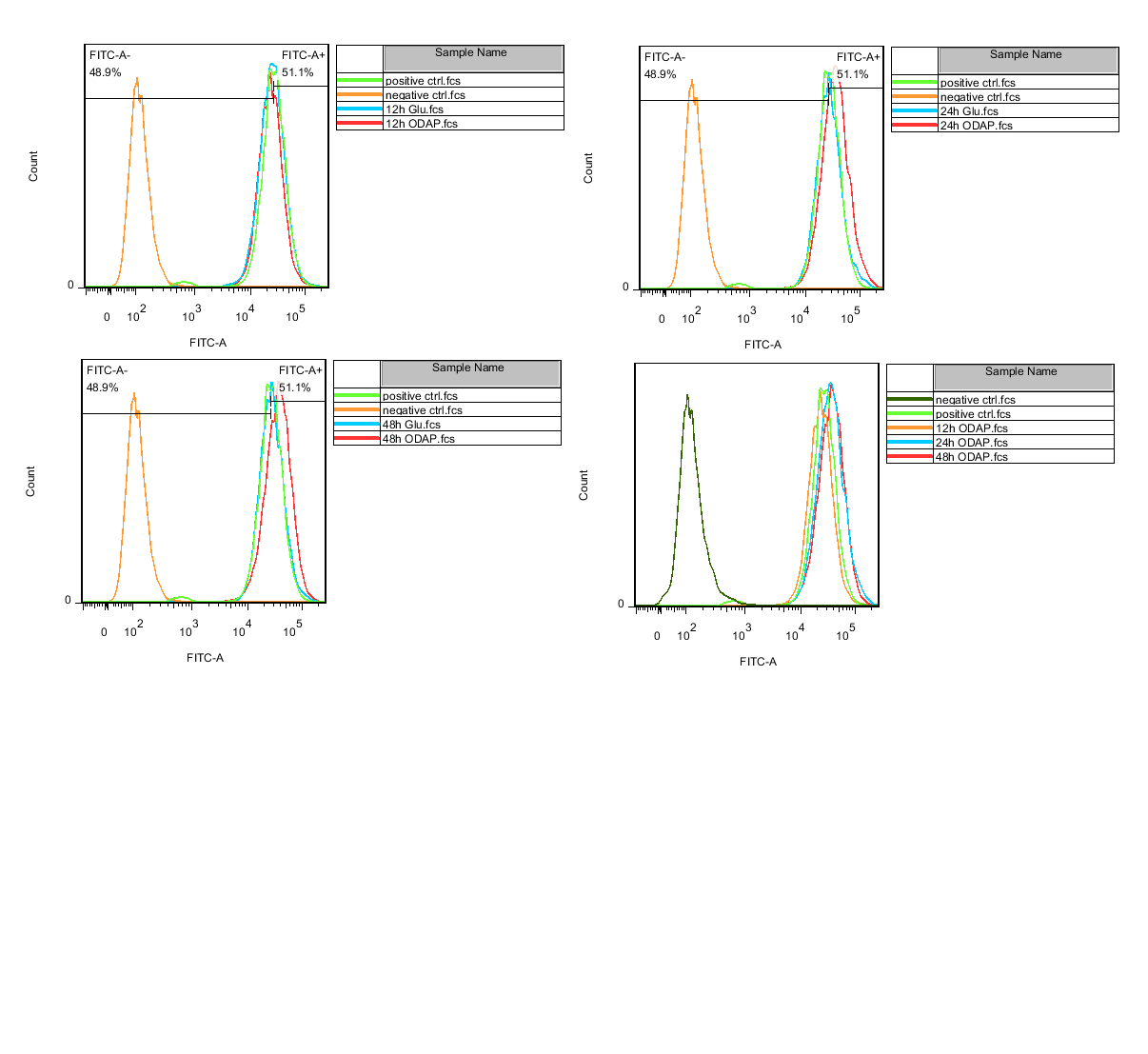


**Fig. S3. Dynamic of mitochondria membrane potential of cells respons to β-ODAP or Glu treatment in 12 h, 24 h and 48 h**
